# Visualization of electrochemically driven solid-state phase transformations using *operando* hard X-ray spectro-imaging

**DOI:** 10.1038/ncomms7883

**Published:** 2015-04-20

**Authors:** Linsen Li, Yu-chen Karen Chen-Wiegart, Jiajun Wang, Peng Gao, Qi Ding, Young-Sang Yu, Feng Wang, Jordi Cabana, Jun Wang, Song Jin

**Affiliations:** 1 Department of Chemistry, University of Wisconsin—Madison, Madison, Wisconsin 53706, USA; 2 Photon Sciences Directorate, Brookhaven National Laboratory, Upton, New York 11973, USA; 3 Department of Sustainable Energy Technologies, Brookhaven National Laboratory, Upton, New York 11973, USA; 4 Advanced Light Source, Lawrence Berkeley National Laboratory, Berkeley, California 94720, USA; 5 Department of Chemistry, University of Illinois at Chicago, Chicago, Illinois 60607, USA

## Abstract

*In situ* techniques with high temporal, spatial and chemical resolution are key to understand ubiquitous solid-state phase transformations, which are crucial to many technological applications. Hard X-ray spectro-imaging can visualize electrochemically driven phase transformations but demands considerably large samples with strong absorption signal so far. Here we show a conceptually new data analysis method to enable *operando* visualization of mechanistically relevant weakly absorbing samples at the nanoscale and study electrochemical reaction dynamics of iron fluoride, a promising high-capacity conversion cathode material. In two specially designed samples with distinctive microstructure and porosity, we observe homogeneous phase transformations during both discharge and charge, faster and more complete Li-storage occurring in porous polycrystalline iron fluoride, and further, incomplete charge reaction following a pathway different from conventional belief. These mechanistic insights provide guidelines for designing better conversion cathode materials to realize the promise of high-capacity lithium-ion batteries.

## Abstract (web summary)

Hard X-ray spectro-imaging using synchrotron radiation can be used to monitor electrochemical reactions. Here, the authors present X-ray absorption data and resolve phase evolution for the conversion of iron fluoride, a high-capacity Li-ion battery conversion cathode, with nanoscale resolution.

## Graphical abstract

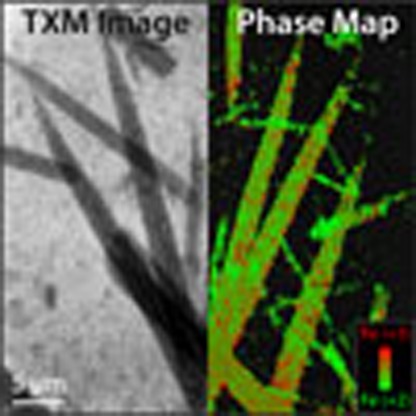

Solid-state phase transformations are ubiquitous and important to numerous applications^1^. They are not only the primary way to make technologically important materials (such as martensitic steel, inorganic ceramics and thin-film absorbers for solar cells), but also lie at the heart of electrochemical energy storage, in which the insertion and extraction of charge storage ions (such as Li^+^ and Na^+^) are always accompanied by electrochemically driven solid-state phase transformations^2^. There has been significant interest in monitoring and probing these transformation processes over the last few decades in the hope of gaining mechanistic understanding to guide further optimization and bring technological benefits. The most commonly used techniques to analyse characteristics of phase transformations include *in situ* X-ray diffraction^3^, neutron diffraction^4^, X-ray absorption spectroscopy (XAS)^5^,^6^ and solid-state nuclear magnetic resonance^6^. These techniques enable valuable insights into changes in long-range structure, local bonding and chemical environment, elemental composition and oxidation state. However, they only reveal average information over a large sample volume (micrometre to millimetre scale). Recently, the remarkable advances in *in situ* transmission electron microscopy (TEM) have made it possible to probe phase transformation down to nanoscale^7^,^8^,^9^,^10^. When coupled with electron diffraction (ED) and/or electron energy-loss spectroscopy, *in situ* TEM serves as a perfect diagnostic tool to track nanoscale morphological and structural changes as well as new phase nucleation and propagation with nanoscale spatial resolution. However, TEM-based experiments require restrictively thin samples (100 nm or below) and must be compatible with the high vacuum environment inside the microscope. These limitations complicate the experimental design and the straightforward correlation of the observed phenomena to what actually occur under realistic conditions. Furthermore, TEM-based techniques are inherently incapable of probing the bulk of a working device, such as a battery or a fuel cell electrode, which is typically made of multiple components assembled at micro-length or larger length scales.

Hard X-ray spectro-imaging provides an innovated approach to visualize electrochemically driven solid-state phase transformations at the nanoscale^11^. Taking advantage of the strong and deeply penetrating hard X-rays generated by synchrotron radiation and the chemical and elemental sensitivity with a full-field imaging capability provided by the transmission X-ray microscopy (TXM) coupled with X-ray absorption near-edge structure spectroscopy (XANES)^12^,^13^, progression of a electrochemical reaction in a realistic battery electrode can be visualized in a large (tens of micrometres) field-of-view (FOV) with nanoscale spatial resolution. Unlike *in situ* TEM experiments that often have to be carried out using specialized sample holders in electrochemical conditions drastically different from those in a practical battery^7^,^8^,^9^, hard X-ray-based experiments can be conveniently performed in relevant and realistic conditions using a much simpler cell design^14^,^15^,^16^,^17^. Proof-of-concept *operando* hard X-ray spectro-imaging experiments have been recently demonstrated by using large microparticles^18^,^19^,^20^,^21^ or aggregates of small particles^20^ (several micrometres in total size) with strong X-ray absorption signal, in which cases chemical phase maps could be generated using simple data processing methods by approximating the X-ray absorption of materials under study to the experimentally determined total X-ray absorption. However, those approaches ignored background absorption and thus cannot accurately determine actual X-ray absorption of smaller and weakly absorbing samples at single-particle level under *operando* conditions. To study small particles, which, in fact, are more electrochemically active and mechanistically relevant, we herein propose and develop a conceptually new data analysis method to reliably determine X-ray absorption of study materials and for the first time realized *operando* studies of phase evolution in a high-capacity Li-ion battery conversion cathode with nanoscale chemical resolution.

Here the conversion reaction of iron fluoride (FeF_3_) was chosen as a demonstration example for *operando* mechanistic studies. FeF_3_ is a promising prototype conversion cathode material with extremely high Li-storage capacity (712 mAh g^−1^)^22^,^23^,^24^,^25^,^26^,^27^,^28^,^29^, four or five times higher than current intercalation cathode materials such as LiCoO_2_ (∼140 mAh g^−1^) and LiFePO_4_ (∼170 mAh g^−1^). This is achieved by utilizing all of the oxidation states of Fe through an electrochemical conversion reaction to enable multiple electron transfer and Li-ion storage.


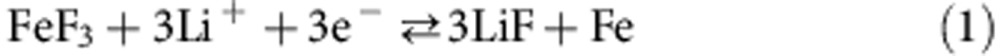


Consequently, batteries based on a FeF_3_ conversion cathode (theoretical voltage ∼2.7 V)^23^ and a suitable lithium-containing anode, such as a protected lithium metal anode^30^, hold the promise to significantly increase the energy density of current Li-ion battery technology^22^,^31^. However, such promise has yet to be realized because of challenges associated with the significant phase transformation and structural rearrangement during cycling. Two prominent ones are capacity decay and a large voltage hysteresis^24^,^26^,^32^,^33^,^34^. Solving these challenges requires a better understanding of the electrochemical reaction mechanism under *operando* conditions, especially the recharge reaction, which has been surprisingly under-researched compared with the discharge reaction^35^,^36^. Here we use the improved *operando* hard X-ray spectro-imaging to track the phase evolution of FeF_3_ cathodes during cycling and reveal how electrochemical reactions progress kinetically and spatially, which provides insights essential to rationally designing electrode microstructure to achieve fast kinetics and high reversibility. We also discover evidences across different length scales that suggest a charge reaction pathway different from the traditional belief, which advances the understanding on the causes of capacity decay and voltage hysteresis.

## Results

### Improved experimental setup and new data analysis method

**Figure 1:**
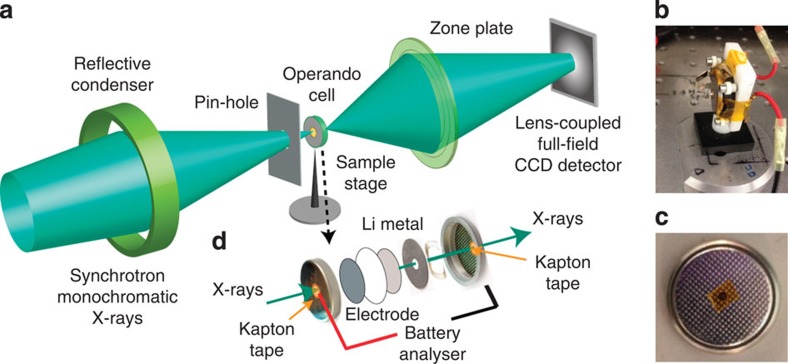
Schematic illustration of the transmission X-ray microscope (TXM) experimental setup. (**a**) Sketch of the full-field TXM. Photographs of (**b**) the custom-built cell holder and (**c**) a perforated coin cell used for the *operando* studies. (**d**) Schematic illustration of the *operando* cell containing the FeF_3_ electrode and all the other key components of a realistic battery.

In the *operando* experiments (Fig. 1a), synchrotron monochromatic X-rays are directed to transmit through a perforated 2032-type coin-cell (Fig. 1b,c) containing the FeF_3_ cathode and all the other key components of a realistic battery, such as carbon black, polymeric binder and a liquid electrolyte (Fig. 1d). The holes in the coin-cell, which are sealed using Kapton tape (Fig. 1c,d), need to be small to ensure a small cell impedance. The resulted absorption-contrast images are projected onto a lens-coupled full-field CCD detector using a zone-plate and recorded.

**Figure 2:**
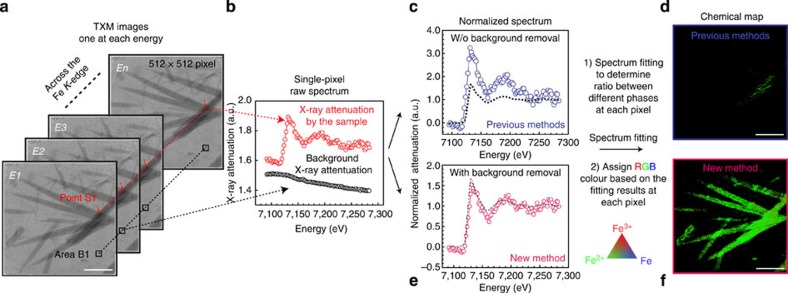
Construction of the chemical phase maps using the new data analysis method. (**a**) A series of TXM image (512 × 512 pixels) collected by scanning across the Fe K-edge, one image at each energy. The *operando* cell was discharged to ∼2.0 V when the data were collected. Scale bar is 10 μm. (**b**) XANES spectra from the areas with and without the study sample, respectively. The background spectrum contains information on X-ray attenuation by all the other components in the *operando* cell. (**c**) XANES spectrum directly normalized without background removal using previously reported methods, which is clearly off the scale compared with a reference spectrum collected from FeF_2_ powder (black dashed line). When the spectra at all pixels are processed the same way and fitted, only very few pixels could be preserved, leading to the unsatisfactory chemical map in **d**. In contrast, **e** shows the spectrum correctly normalized by subtracting the background X-ray attenuation first before the normalization. The reference spectrum collected from FeF_2_ powder (black dashed line) is shown as a comparison. (**f**) High-quality chemical phase maps constructed by fitting the background-subtracted normalized spectra at each pixel.

Figure 2 illustrates general procedures to realize chemical phase mapping; a detailed comparison between previous methods (Fig. 2a–d) and our new method (Fig. 2a, b, e, f) are discussed later in the manuscript. First, a series of images are collected by scanning the energy across the Fe *K*-edge (7,112 eV) in a step size of 2 eV, one image at each energy (Fig. 2a). Then, the XANES spectra at each pixel are constructed by plotting normalized X-ray absorption versus energy (Fig 2b,c, or b,e). They are fitted to reference spectra collected from standard compounds (Fe^3+^F_3_, Fe^2+^F_2_ and Fe) to determine ratio between different Fe-containing phases so that red-green-blue (RGB) colours can be accordingly assigned to generate a phase map (Fig 2d or f). As Fe of various oxidation states interact with X-ray differently, their spatial distribution at different states of discharge/charge reveals progression of the electrochemical reaction in the FeF_3_ cathode.

We have developed a conceptually new data analysis method to enable *operando* spectro-imaging of small samples with weak absorption signal (see Methods and more details available in Supplementary Methods). In *operando* experiments, the X-rays are not just absorbed by the Fe-containing-active material, but also attenuated by all other battery components in the pathway of the beam, such as the carbon black, polymeric binder, separator and liquid electrolyte, which may be considered as an internal background as a whole. Previous methods depend heavily upon strong X-ray absorption of large samples (tens of micrometres in size) and consider that the X-ray absorption of materials under study is approximately equal to the experimentally determined total X-ray absorption^18^,^19^,^20^,^21^, in which background absorption is omitted. This approximation is no longer valid for smaller and weakly absorbing samples, such as the porous microwires (MWs) examined here (effective thickness <1 μm when porosity is considered). It results in improperly normalized XANES spectra (Fig. 2c) and consequently unsatisfactory chemical phase maps (Fig. 2d and see an example of complete comparison in Supplementary Fig. 1). We propose and show that the X-ray absorption of the Fe-containing-active material can be determined more accurately by subtracting an internal background spectrum from the total X-ray attenuation over the whole imaging area of 512 × 512 pixels. We use the X-ray attenuating information readily available from an area in the same FOV, where the X-rays only pass through the other battery components but not the Fe-containing-active material (Fig. 2a, black box), to more accurately represent the internal background absorption spectrum (Fig. 2b, black circles). We then subtract such background from the total X-ray absorption spectrum (Fig. 2b, red circles) at each pixel to determine the actual X-ray absorption of the study material. We also note that background absorption can be mathematically fitted to allow high-quality normalization within a single XANES spectrum^37^,^38^, but such methods are extremely calculation-intensive to implement when dealing with a considerably large number of spectra (512 × 512 spectra) in spectro-imaging. After internal background removal using our new method, the XANES spectrum (Fig. 2e) at each pixel can be correctly normalized and fitted to a linear combination of standard reference spectra (Supplementary Fig. 2), enabling high-quality chemical phase mapping under *operando* battery conditions (Fig. 2f). Note that even though the intrinsic spatial resolution is dictated by the current instrumentation (∼25 nm for camera binning 1)^13^, the new data analysis method helps determine the X-ray absorption of weakly absorbing samples more accurately at single-particle level so that many more image pixels that contain meaningful chemical information are preserved. These improvements enable the chemical phase mapping of samples that are smaller in size (hundreds of nanometres) than those reported before (several micrometres in total size)^18^,^19^,^20^,^21^.

### FeF_3_ model samples and *in situ* electrochemical cell

**Figure 3:**
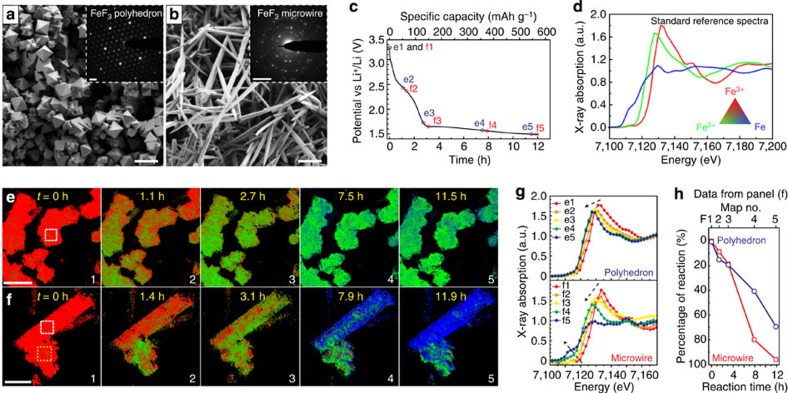
Visualization of the discharge reaction of FeF_3_ cathodes using hard X-ray spectro-imaging. (**a**,**b**) Scanning electron micrographs of the FeF_3_ polyhedra and MWs. Insets are ED patterns taken on an individual polyhedron and an individual MW showing that the polyhedron is single-crystalline, whereas the MW is polycrystalline. Scale bars are 10 μm in the two panels and 5 nm^−1^ in their insets. (**c**) Discharge voltage profile of the *operando* cell that contains a FeF_3_ cathode of mixed polyhedra and MWs. The cell was discharged at rate of ∼1/15 C to 1.5 V. The small black circles indicate the states of discharge where the data were collected to construct chemical phase maps. (**d**) Reference spectra collected from standard compounds (Fe^3+^F_3_, Fe^2+^F_2_ and Fe). (**e**) and (**f**) are two series of chemical phase maps showing how the electrochemical discharge reaction proceeded in two different regions of the mixed FeF_3_ electrode. Scale bars are 10 and 5 μm, respectively. Red, green and blue pixels represent Fe (+III), Fe (+II) and metallic Fe, respectively. (**g**) Two series of XANES spectra taken from two selected areas of the mixed electrode, indicated by the white boxes in the first maps of **e** and **f**, which show that the discharge reaction of the MW was more complete than that of the polyhedron. This is further confirmed by a quantitative comparison in reaction progress (consumption of FeF_3_) between a MW and a polyhedron (**h**). The data were taken from two selected areas indicated by the white and yellow boxes in the first map of **f**.

Our *operando* experiments also benefit from two specially designed FeF_3_ samples with well-defined morphologies of polyhedron (Fig. 3a) and MWs (Fig. 3b). These two samples are synthesized for the first time by rationally controlling supersaturation^26^,^39^,^40^,^41^ (see Methods for synthetic details) and are quite different in microstructure, porosity and electrochemical activity. The FeF_3_ polyhedra are single-crystalline (as proven by the ED pattern in Fig. 3a inset), non-porous (Supplementary Fig. 3) and can only reach approximately one-third of the theoretical Li-storage capacity in the conventional battery test (Supplementary Fig. 5), whereas the MWs are polycrystalline (Fig. 3b inset), mesoporous, grain-boundary-rich (Supplementary Fig. 4) and almost reach full capacity (Supplementary Fig. 5). We made an electrode containing both the polyhedra and MWs to enable comparative study and reveal the relation between structure and electrochemical properties. Porous carbon paper (∼110 μm in thickness) was used as the current collector for this electrode. It is quite transparent to hard X-rays but still robust enough for handling, which is critical to the *operando* spectro-imaging experiment. More details on the electrode and cell preparation can be found in Supplementary Methods. The *operando* cell was discharged at a constant current of ∼1/15 C (∼47.5 mA g^−1^) to 1.5 V (Fig. 3c). TXM images were recorded in dynamic conditions at different states of discharge/charge in two different locations. The data were processed by our new approach and fitted to the standard reference spectra (Fig. 3d) to generate the chemical phase maps shown in Fig. 3e (FOV 1) and 3f (FOV 2). Similar *operando* studies were also carried out in an electrode containing only FeF_3_ MWs cycled in potentiostatic mode (Supplementary Fig. 6).

### *Operando* visualization of FeF_3_ electrochemical reaction

Enabled by the technological advances in spectro-imaging, we first visualized the progression of electrochemical discharge reaction in the FeF_3_ conversion cathode. Two consecutive phase transformations consistent with the sequential lithiation reaction of FeF_3_ (refs 26, 32, 33) were observed in both the polyhedra and MWs (Fig 3e,f). First, from the open-circuit voltage 3.24 to 1.62 V, red pixels were gradually replaced by green pixels (Fig 3e,f, map 1 to 3), which is related to the initial Li^+^ intercalation into FeF_3_ with Fe (+III, red colour) being reduced to Fe (+II, green colour). Then, in the sloping voltage plateau between 1.62 and 1.5 V, blue pixels appeared at the expense of the green pixels (Fig 3e,f, map 4 to 5), indicating the formation of metallic Fe (blue colour) through the conversion reaction. However, although the first reduction could proceed to completion in both FeF_3_ samples, the progression of the second reduction is clearly quite different. The conversion from Fe (+II) to metallic Fe was incomplete for the polyhedra (Fig. 3e, map 5). Compared with the reference spectra (Fig. 3d), selected-area XANES spectra taken on the polyhedron reveals that Fe (+II) and metallic Fe co-existed in the end (Fig. 3g, top panel). The contrast in electrochemical activity is further highlighted in Fig. 3f, in which one MW and one polyhedron are situated side by side. The MW became mostly metallic Fe (mostly blue), whereas the polyhedra did not react completely (still a lot of green) at the end of discharge (Fig. 3f, map 5). The change in XANES spectra taken on the MWs (Fig. 3g) is consistent with the successive reduction of Fe (+III) to Fe (+II) and then to metallic Fe. Quantitative comparison of the reaction progress based on spectrum fitting (Fig. 3h) also confirms that the MWs react faster and more completely than the polyhedra. These observations not only explain the difference in discharge capacity in our conventional battery tests (Supplementary Fig. 5) but also reveal the importance of porous and grain-boundary-rich structure to promote the complete three-Li^+^ storage for FeF_3_ conversion cathodes. During discharge, electron transport may become less of a concern once metallic iron precipitates out and starts propagating to form a conductive network within the solid^8^,^26^,^35^,^42^. Therefore, the key to achieving high capacity likely depends on efficient Li^+^ transport to trigger the Fe and LiF precipitation after the structure is saturated with Li^+^ (ref. 8). This process could be facilitated by the porous and grain-boundary-rich structure of the MWs.

Furthermore, we were able to visualize the spatial dynamics of the electrochemical reaction over a large area (tens of micrometres) thanks to the unique capability of TXM-XANES in spatially resolving chemical identification in a large FOV. As shown by the concurrent colour change in the ensemble of polyhedra (Fig. 3e) and MWs (Supplementary Fig. 6), the electrochemically driven phase transformations in the FeF_3_ cathode are relatively homogenous, indicating the absence of preferential reaction sites during discharge. All particles were actively discharging, although some local regions on individual particles appeared to react more slowly. Because these experiments were carried out on both single-crystalline and polycrystalline FeF_3_ samples under *operando* conditions, we believe this observation reveals the inherent reaction characteristics of FeF_3_ conversion cathodes. Interestingly, this behaviour is different from that of the LiFePO_4_ intercalation cathode material, in which significant inhomogeneities (that is, active particle fraction<100%) were observed and believed to be a major limiting factor for further improving high-rate performance^43^,^44^. Therefore, this *operando* visualization of FeF_3_ (active particle fraction=100%) suggests that it could be feasible for conversion cathode material to achieve better rate capability than what was traditionally believed^22^,^24^, likely via further nanostructure engineering^26^.

**Figure 4:**
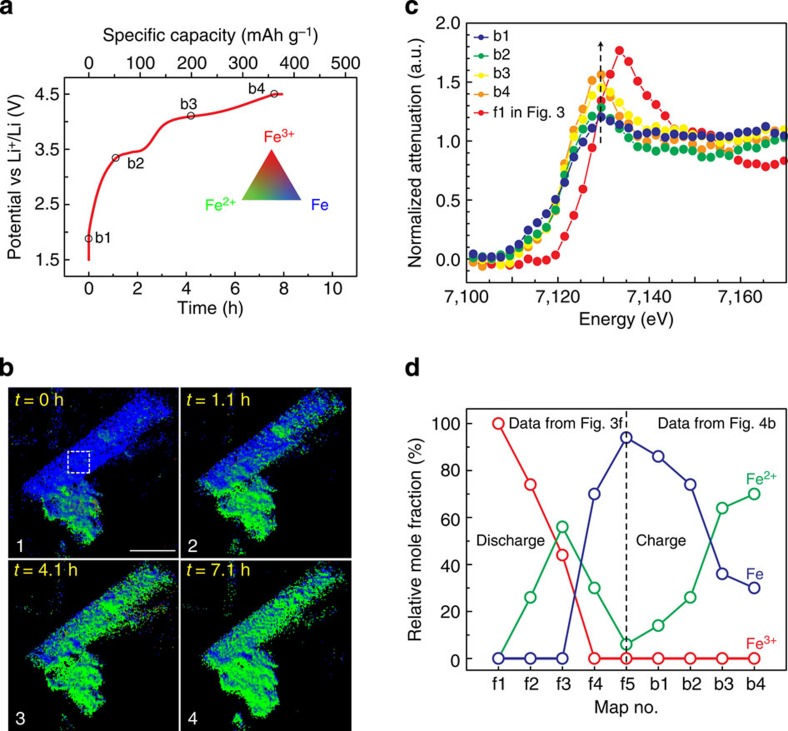
Visualization of the charge reaction of FeF_3_ cathodes using hard X-ray spectro-imaging. (**a**) Voltage profile of the *operando* cell that was recharged at ∼1/15 C to 4.5 V. The small black circles indicate the states of charge where the data were collected to construct the series of chemical phase maps in **b**, which show the progression of electrochemical reaction during charge. Scale bar is 5 μm. (**c**) XANES spectra taken from a selected area of the MW, marked by the white box in the first maps of **b**, showing the change in X-ray absorption during charge. The XANES spectrum of the pristine FeF_3_ electrode is shown for comparison. (**d**) The mole fraction of the Fe species in different oxidation states in the same selected area (indicated by the white box) of map 1 to 5 of Fig. 3f (discharge) and map 1 to 4 of panel **b** (charge), determined by linear combination fitting using reference spectra.

Our *operando* experiments further enabled the investigation of the charge reaction of the FeF_3_ cathode. This unique charge (delithiation) study has not been possible so far using *in situ* TEM techniques due to the instability of organic electrolyte under electron beam and/or the difficulty of applying a controlled constant current. The *operando* cell was charged at the same rate of ∼1/15 C to 4.5 V (Fig. 4a) and chemical phase maps at four different states of charge are shown in Fig. 4b. The electrochemical reaction occurred uniformly everywhere in the sample, similar to what was observed during discharge. The overall phase transformation during charge is in agreement with metallic Fe gradually converting into Fe (+II) as the voltage increases, but not reaching Fe (+III). This can be clearly seen from the chemical phase maps as well as their corresponding XANES spectra (Fig. 4c) taken from a selected area of the MW. The relative mole fraction of different Fe oxidation states during both discharge and charge was determined by spectrum fitting and shown in Fig. 4d. When the charge process finished at the cutoff voltage of 4.5 V, the dominant oxidation state was Fe (+II) (∼70%, Fig. 4d), which is consistent with what was suggested by the previous *ex situ* TEM-electron energy-loss spectroscopy studies on the recharged FeF_3_/C electrodes^45^. There was also some metallic Fe that did not react during recharge (∼30%, Fig. 4d). The *operando* studies in voltammetric mode showed the same phase transformation behaviour (Supplementary Fig. 6).

### Phase transformations over a large area of the electrode

**Figure 5:**
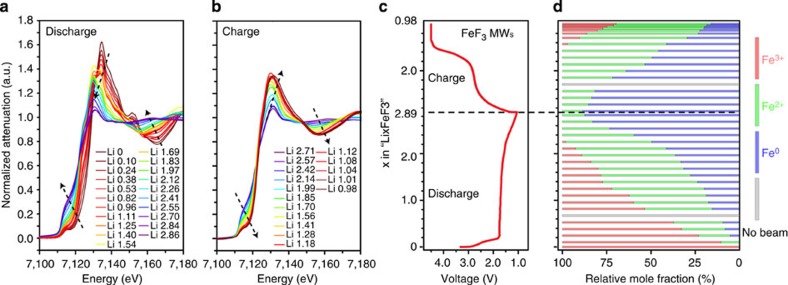
*Operando* XAS on a FeF_3_ MW cathode. XANES spectra collected at different states of discharge (**a**) and charge (**b**). In **a** and **b**, the same vertical axis is shared. The arrows indicate the changing trend in XANES spectra during discharge and charge. (**c**) Discharge and charge voltage profile of the *operando* cell cycled at a rate of ∼1/12 C. After the constant-current charging, a constant-voltage charging step was applied until the current dropped to ∼1/50 C. (**d**) The mole fraction of the Fe species in different oxidation states at different states of discharge and charge, which is calculated by linear combinational fitting of the XANES spectra. In **c** and **d**, the same vertical axis is shared.

To confirm what we observed locally in the two FOVs represents the global changes that occurred in the whole electrode, we collected additional data from a few other areas at the end of discharge and charge. The resulting chemical phase maps give consistent pictures (Supplementary Fig. 7). Furthermore, we carried out an *operando* XAS experiment on a FeF_3_ MW electrode cycled at ∼1/12 C rate. The larger size X-ray beam (∼0.5−1.0 mm) in the spectroscopy experiment allows the tracking of phase transformation over a much larger area of the electrode. The observed changes in XAS spectra collected at different states of discharge (Fig. 5a) and charge (Fig. 5b) during continuous cycling (Fig. 5c) are in agreement with what we observed locally in a single MW (Figs 3g and 4c) and the corresponding chemical phase maps (Figs 3f and 4b), indicating that the electrochemical reaction is relatively homogeneous over a large area of the electrode. We further performed spectrum fitting to determine the ratio between different Fe oxidation states during cycling (Fig. 5d). Fitting details and representative best fits are shown in Supplementary Table 1 and Supplementary Fig. 8, respectively. The fitting result corroborates our findings in the spectro-imaging experiments (Fig. 4d). The charge reaction went through Fe (+II) containing intermediate phases (green bar in Fig. 5d) and Fe (+III, red bar in Fig. 5d) was barely recovered when recharged to 4.5 V, but could indeed be formed if a constant-voltage charging step was applied following the constant-current charging. *Ex situ* XAS spectra (Supplementary Fig. 9) were also collected from electrodes that were cycled to different states of discharge/charge. The result is consistent with those obtained under *operando* conditions.

## Discussion

Our investigation of the charge reaction across different length scales provides a plausible explanation for the first cycle capacity loss, which is a problem commonly observed for FeF_3_ cathodes^24^,^26^,^32^ but poorly understood. We found that the capacity loss was caused by the incomplete reconversion of Fe to Fe (+III) during charge, which appears to be a kinetically limited process considering the slow diffusion of iron and fluoride ions. Another important implication is the indication of a different charge reaction pathway from the previous understanding. First-principles calculations previously suggested that a number of Fe (+III) containing compounds form sequentially during charge, which seemingly offered a plausible explanation for the voltage hysteresis between discharge and charge^33^. However, the *operando* investigation herein shows Fe (+II)-containing compounds formed first and Fe (+II) was slowly converted to Fe (+III) at high voltage during charge. A thorough (re)investigation using integrated experimental and theoretical approaches would be useful to address this discrepancy between our results and the previous simulation.

We have developed an *operando* X-ray spectro-imaging technique empowered by a new data analysis method to more accurately account for the internal background absorption and determine the X-ray absorption of weakly absorbing samples to enable the first visualization of the electrochemical reaction in high-capacity FeF_3_ cathodes at the nanoscale. These studies reveal the importance of porous nanostructure in achieving fast conversion reaction kinetics and high capacity for FeF_3_ cathodes, which could be a structural design principle generally applicable to other conversion electrode materials^22^. Further, more research efforts should focus on the charge reaction, which appears to be kinetically slow even for a highly active porous nanostructured FeF_3_ cathode and is confirmed as the bottleneck to utilize the full capacity of conversion electrode materials upon cycling. This work also provides guidelines in experimental design of *in situ* electrochemical cell fabrication, background subtraction and data analysis method to facilitate *operando* mechanistic studies of other electrode materials^22^,^46^,^47^ to elucidate reaction pathways and diagnose possible failure mechanisms. The temporal and spatial resolution of the chemical phase mapping will be further improved with the full commissioning of brighter synchrotron light sources, together with the development of lensless imaging methods^48^ and better data processing algorithms. This will ultimately allow *operando* studies of other complex solid-state phase transformations that are not electrochemically driven and lead to beneficial solutions towards many technological applications.

## Methods

### Synthesis of FeF_3_ MWs and polyhedra

FeF_3_ MWs were prepared by thermal dehydration of *α*-FeF_3_·3H_2_O MWs at 350 °C for 2.5 h in argon atmosphere. The precursor α-FeF_3_·3H_2_O MWs were synthesized by reacting Fe(NO_3_)_3_·9H_2_O and HF aqueous solution in ethanol with a concentration ratio of *c*(Fe^3+^):*c*(HF):*c*(H_2_O) ≈53.2 mM:500 mM:11,575 mM at 60 °C for 18 h. Rhombohedral phase FeF_3_ polyhedra were prepared by thermal conversion of the metastable cubic phase FeF_3_ polyhedra at 350 °C for 30 min in argon atmosphere. The precursor cubic phase FeF_3_ polyhedra were synthesized by reacting Fe(NO_3_)_3_·9H_2_O and HF aqueous solution in ethanol with a concentration ratio of *c*(Fe^3+^):*c*(HF):*c*(H_2_O) ≈ 20 mM:100 mM:4,670 mM at 60 °C for 24 h. More synthetic details can be found in the Supplementary Methods.

### Characterization

Scanning electron microscopy images were acquired using a LEO 55 VP scanning electron microscope at 5 kV. TEM images and ED patterns were acquired using either a Tecnai T-12 (120 kV) or a FEI Titan TEM (200 kV). Powder X-ray diffraction data were collected on a Bruker D8 diffractometer using Cu K*α* radiation. The Brunauer–Emmet–Teller surface area and pore size distribution of the FeF_3_ MWs were calculated from nitrogen adsorption–desorption isotherms measured by a Quantachrome Autosorb-1 gas sorption analyser. *Ex situ* electrochemical measurements were performed on electrodes made of 70 wt% active material, 20 wt% carbon black and 10 wt% binder. CR2032-type coin cells were assembled in an argon-filled glovebox, using Li metal as the counter/quasi-reference electrode, 1 M LiPF_6_ in EC/DMC (1/1 by volume) as the electrolyte and electrolyte-soaked polyethylene-polypropylene films as the separator. Electrochemical impedance spectroscopy and galvanostatic cycling were performed using either a Biologic SP-200 or a VMP-3 Potentiostat/Galvanostat controlled by EC-Lab software.

### *Operand*o hard X-ray spectro-imaging and chemical map construction

The *operando* hard X-ray spectro-imaging experiments were performed using the full-field TXM at beamline X8C, National Synchrotron Light Source, Brookhaven National Laboratory, using a perforated 2032-type coin cell with holes on both sides sealed by Kapton tapes. The holes were sealed using Kapton tapes and need to be small to ensure a small cell impedance. The o*perando* measurements were performed on electrodes made of 30 wt% FeF_3_-active material (polyhedra and MWs 1:1 by weight, or MWs only), 50 wt% carbon black and 20 wt% binder (see a representative scanning electron microscopy image in Supplementary Fig. 10). Note that both carbon black and binder are transparent to hard X-rays. Thin aluminum foils (∼8 μm thickness) or carbon papers (∼110 μm thickness) were used as current collectors for the FeF_3_ electrodes. The cell was put into a custom-built holder mounted on a motorized *X, Y, Z, θ* stage and aligned so that the X-ray beam could transmit through. A FOV of 40 × 40 μm^2^ with a 2,048 × 2,048 CCD camera was used. The cell was continuously cycled in galvanostatic or potentiostatic mode and absorption-contrast images (X-ray transmitted through the sample) and reference background images (X-ray passing through air) were collected in sequence under dynamic conditions. To track the phase transformations in the electrode, a full series of TXM images were collected at each state of discharge and charge. Each TXM image series was collected by scanning across the Fe *K*-edge (7,112 eV) from 7,091 to 7,285 eV, with a step size of 2 eV and taking one TXM image at each energy step, which contains 512 × 512 XANES spectra when using 4 × 4 binned camera binning. The exposure time for each image was 4 s. Each chemical phase map took ∼8 min to finish. After collection each set of data, the area of study (FOV 1) was allowed to rest for ∼16 min (not exposed to X-rays) to minimize any potential impact induced by the X-ray beam, during which a new set of data was taken in another area of study (FOV 2) and background reference images (X-ray passing through air) were also recorded after that. The output pixel size is ∼80 nm (camera binning 4).

The XANES spectrum at each pixel was normalized using our new method and then fitted with the linear combination of standard reference spectra collected from FeF_3_, FeF_2_ and Fe powders under the same conditions using TXM. The rutile FeF_2_ was used to represent all the possible rutile-related Fe^2+^-containing phases. This is a reasonable approximation because it was reported that the Li_*x*_FeF_3_ (when *x*≈1.0) phase contains structural features that are found in the rutile FeF_2_ structure^23^. The spectrum fitting was carried out by minimizing the ***R*** value (a measure of misfit) for each spectrum at each pixel, which is defined as:


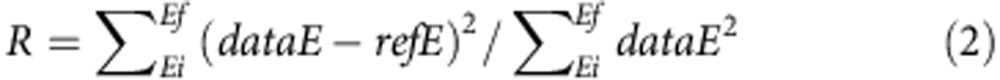


where ***Ei*** is 7,091 eV, ***Ef*** is 7,285 eV, ***dataE*** is the normalized spectrum at each pixel for the given energy ***E***, and ***refE*** is the possible fitting reference value that is a linear combination of X-ray attenuation of FeF_3_, FeF_2_ and Fe. *R values* were minimized at each pixel to find the best-matched phase combination of different Fe oxidation states so that Red–Green–Blue (Red: Fe^3+^, Green: Fe^2+^, Blue: Fe) colours can be assigned accordingly to generate the chemical phase maps. We applied an *R-value* filter (misfit filter) to the resulting phase map and only pixels with *R*<0.08 were displayed in order to give the most accurate chemical phase information. See full details in Supplementary Methods.

### *Operando* XAS

The *operando* X-ray absorption experiments were performed at beamline X18A, National Synchrotron Light Source, Brookhaven National Laboratory, using a perforated 2032-type coin cell with holes on both sides sealed by Kapton tapes. The measurements were performed on electrodes made of 70 wt% FeF_3_-active material, 20 wt% carbon black and 10 wt% binder. The measurements were performed in transmission mode using a Si (111) double-crystal monochromator, which was detuned to ∼35% of its original maximum intensity to eliminate the high order harmonics in the beam. A reference X-ray absorption spectrum of Fe (*K*-edge 7,112 eV) was simultaneously collected using a standard Fe foil. Energy calibration was done using the first inflection point of the Fe *K*-edge spectrum as the reference point. The X-ray absorption data were processed and analysed using IFEFFIT-ATHENA^37^. Standard reference spectra from FeF_3_, FeF_2_ and Fe powders were collected to carry out spectrum fitting and determine the ratio between different Fe oxidation states. *Ex situ* spectra were also collected from electrodes cycled to different states of discharge and charge. The electrodes were recovered from coin cells disassembled in the glovebox and sealed in between two pieces of Kapton tape.

## Author contributions

L.L, Y.-S.Y, J.C., J.W. and S.J. designed the *operando* X-ray spectro-imaging experiments. L.L, Y.-c. K. C.-W. and J.J.W. performed the spectro-imaging experiments. L.L and Y.-c. K. C.-W. developed the new data-analysis method and analysed the chemical mapping results. L.L, P.G. and F.W. designed and performed the *operando* X-ray absorption spectroscopy experiments. L.L performed the material synthesis, *ex situ* structural characterizations and electrochemical tests. Q.D. assisted with the structural characterizations. L.L and S.J. wrote the manuscript with inputs from other co-authors.

## Additional information

**How to cite this article:** Li L. *et al*, Visualization of electrochemically driven solid-state phase transformations using *operando* hard X-ray spectro-imaging. *Nat. Commun*, 6:6883 doi: 10.1038/ncomms7883 (2015).

## Supplementary Material

Supplementary Information Supplementary Figures 1-10, Supplementary Table 1, Supplementary Methods and Supplementary References
