# Supplementary material for: Visualization of electrochemically driven solid-state phase transformations using operando hard X-ray spectro-imaging
Source: Nat Commun. 2015 Apr 20;6:6883. doi: 10.1038/ncomms7883 (PMC4411298; doi:10.1038/ncomms7883)
Supplement: Supplementary Information — Supplementary Figures 1-10, Supplementary Table 1, Supplementary Methods and Supplementary References [file ncomms7883-s1.pdf]

Supplementary Figures

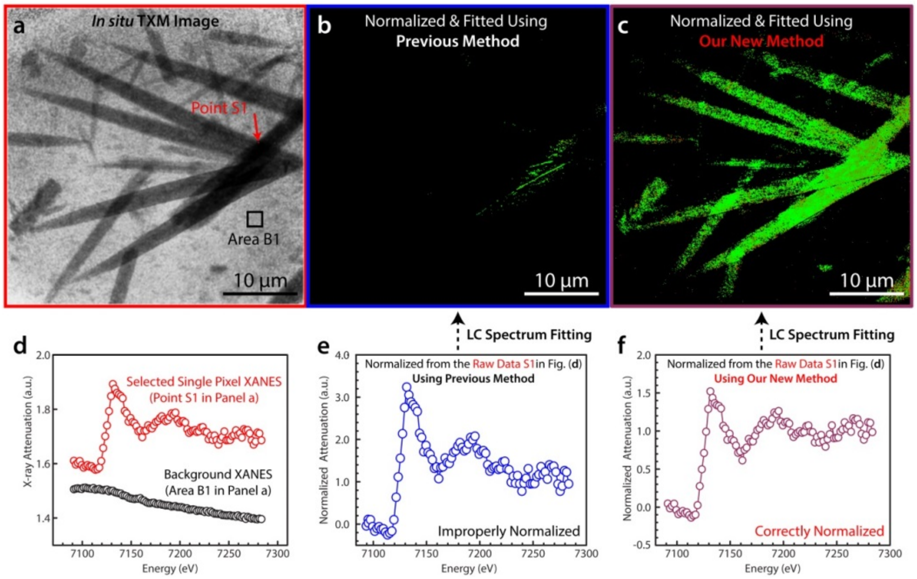

Supplementary Figure 1 | Construction of chemical phase maps using improved data processing method. (**a**) Absorption-contrast image shown along with the chemical phase maps constructed using different data process approaches (**b**, **c**) for the same region in a FeF_3 MW electrode. We took XANES spectra from the areas with and without the FeF_3 MWs respectively and used them as examples to demonstrate the difference of data processing approaches in generating chemical phase maps. The randomly selected single pixel XANES spectrum from the sample-containing area and the background spectrum are shown in (**d**). The background spectrum contains information on X-ray attenuation by all the other components in the *operando* cell, such as electrolyte, polymeric binder, carbon black, current collector, and separator. (**e**) shows the spectrum directly normalized using previously reported methods[4–7], which is clearly off the normal scale (typically 0–2 in the y-axis) compared with the reference standard spectra in **Supplementary Figure 2**. When all the other spectra at other pixels were treated the same way and fitted, only very few of them could be preserved to generate the unsatisfactory phase map in (**b**). In contrast, (**f**) shows the spectrum correctly normalized and scaled using our new approach. The background X-ray attenuation was subtracted first before the normalization. High-quality chemical phase maps, such as that shown in (**c**), can then be generated by fitting the background subtracted normalized spectra at each pixel.

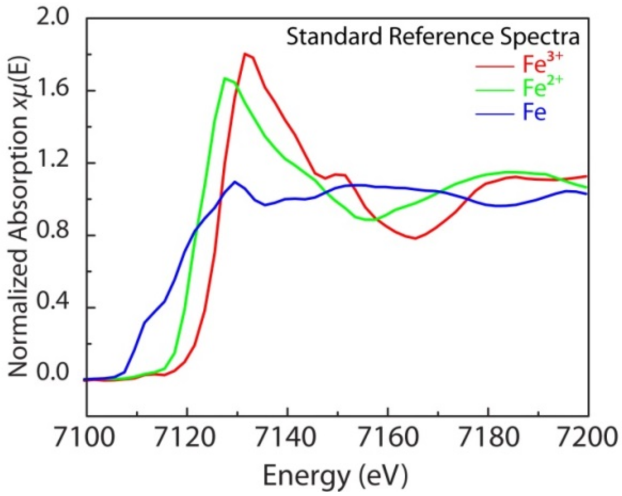

Supplementary Figure 2 | Reference spectra used for fitting to generate chemical phase maps. XANES spectra were collected from commercial $Fe^{3+}F_3$, $Fe^{2+}F_2$, and metallic Fe powders using TXM-XANES.

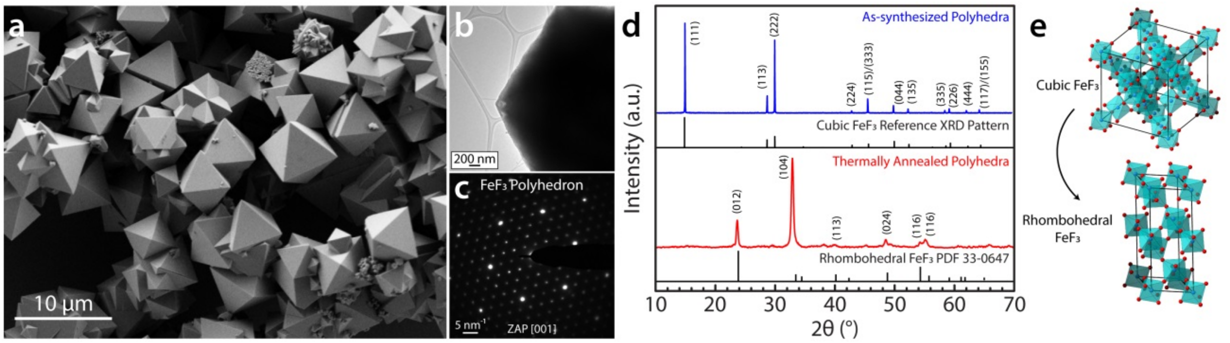

Supplementary Figure 3 | Complete structural characterization of the FeF_3 polyhedra. (**a**) SEM image of the FeF_3 polyhedra with rhombohedral crystal structure. (**b**) Representative TEM image of a polyhedron. (**c**) Selected-area electron diffraction pattern taken from the same polyhedron in (**b**), showing that the polyhedron is single-crystalline. (**d**) Powder X-ray diffractograms of the polyhedra before and after thermal treatment in comparison with the reference standard PDF cards. The as-made polyhedra are phase-pure cubic FeF_3 and they are fully converted to rhombohedral FeF_3 after the thermal treatment in argon at 350 °C for 30 min. (**e**) shows the crystal structures of cubic and rhombohedral FeF_3. The conversion from the cubic phase to the rhombohedral phase involves rotation and distortion of the corner-sharing FeF_6 octahedra. The crystallographic information of cubic FeF_3 was obtained from the Inorganic Crystal Structure Database (ICSD). Note that rhombohedral FeF_3 is the thermodynamically stable phase.

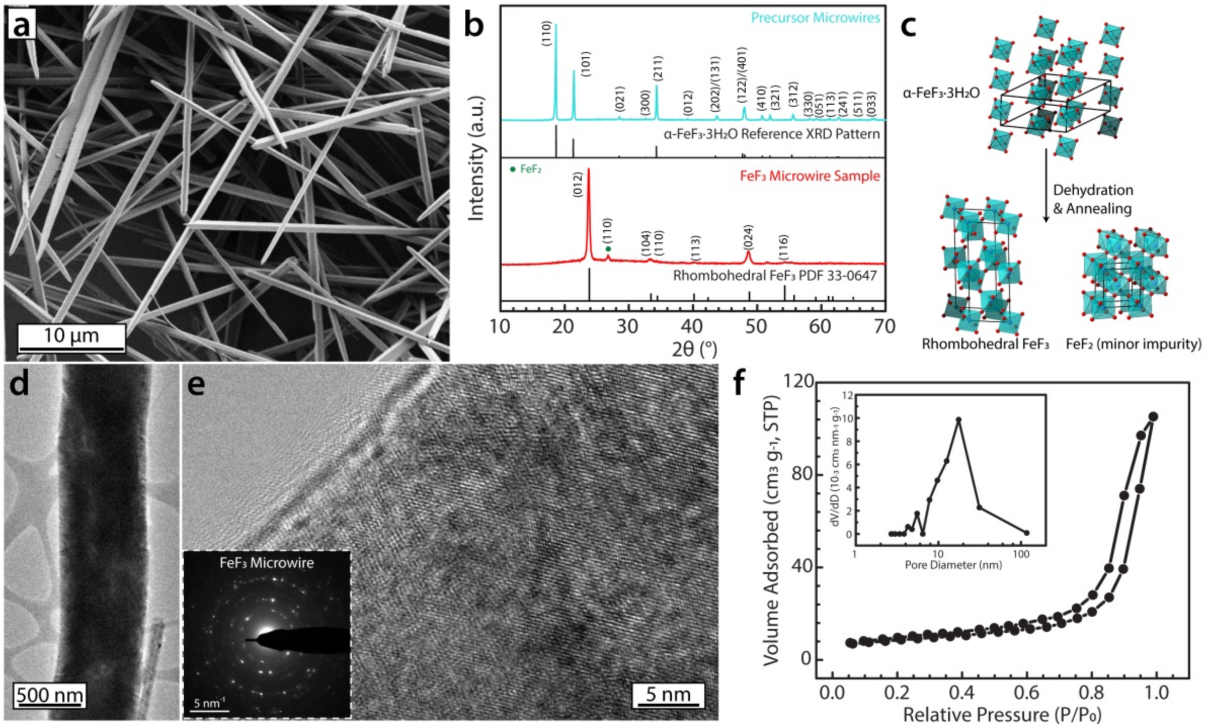

Supplementary Figure 4 | Complete structural characterization of the FeF_3 MWs. (**a**) SEM image of the FeF_3 MWs. (**b**) Powder X-ray diffractograms of the MWs before and after thermal conversion in comparison with the reference standard PDF cards. The thermal dehydration of α-FeF_3·$3H_2O$ MWs led to the formation of MWs of rhombohedral FeF_3. FeF_2 was also produced as a minor impurity phase during the dehydration. (**c**) shows the crystal structures of α-FeF_3·$3H_2O$, FeF_3, and FeF_2. α-FeF_3·$3H_2O$ is made of separate $FeF_3(H_2O)_3$ octahedra with F^- and H_2O randomly distributed. FeF_3 and FeF_2 are made of FeF_6 octahedra connected by sharing corners or edges. The dehydration and conversion of α-FeF_3·$3H_2O$ involves the loss of H_2O and reorganization of the octahedra. (**d**) Low-magnification TEM image of a FeF_3 wire, suggesting porosity. (**e**) High-resolution TEM image of the FeF_3 wire shown in (**d**) showing that the wire is polycrystalline and contains many grain boundaries. The inset is a selected-area electron diffraction pattern taken from the wire in (**d**). The diffraction ring pattern can be indexed to rhombohedral FeF_3 and confirms the polycrystallinity of the wire. (**f**) Nitrogen adsorption/desorption profile of the FeF_3 MWs, revealing that the MWs are mesoporous. The inset shows the pore size distribution. The BET surface area is ~30 m^2/g and the mode (most frequent value) of the pore size is ~17 nm.

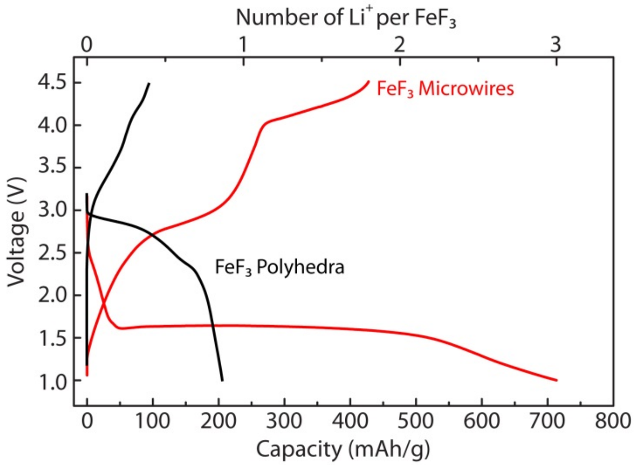

Supplementary Figure 5 | Electrochemical capacity tests of the FeF_3 polyhedra and MWs packed in coin-cell batteries. The FeF_3 polyhedra and MWs were mixed with carbon black and polymeric binder in a 7:2:1 weight ratio and packed into two different 2032-type coin cells for testing. The discharge/charge current was set at 50 mA/g (~1/14.2 C, 1C = 712 mA/g).

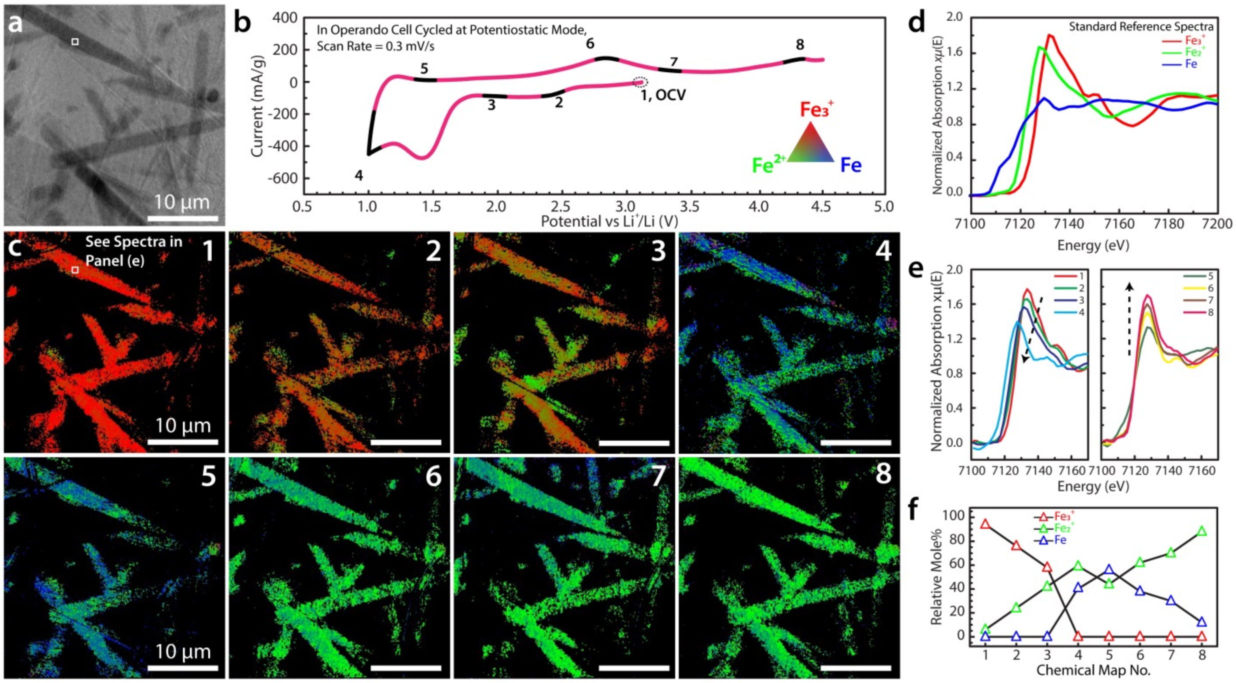

Supplementary Figure 6 | TXM-XANES visualization of the electrochemical reaction of FeF_3 microwire electrode in potentiostatic mode. (**a**) Absorption-contrast image of the area of analysis. (**b**) Voltammogram of the *operando* cell at voltage scan rate of 0.3 mV/s. The black segments on the voltammogram indicate the time periods when the XANES data were collected. (**c**) Reference standard spectra collected from commercial FeF_3, FeF_2, and Fe powders. (**d**) Chemical phase maps at different states of discharge/charge. Conversion from Fe^{3+} to Fe^{2+} and Fe can be clearly seen in (Panel **c** 1–4). Reconversion from Fe went through Fe^{2+}-containing phases and did not go back to Fe^{3+} (Panel **c** 5–8). (**e**) Selected-area XANES spectra to show the changes during the electrochemical conversion/reconversion. The white squares in (**a**) and (**c**) mark the area used to generate the XANES spectra. (**f**) The mole percentage of the Fe species in different oxidation states in map c1 to c8.

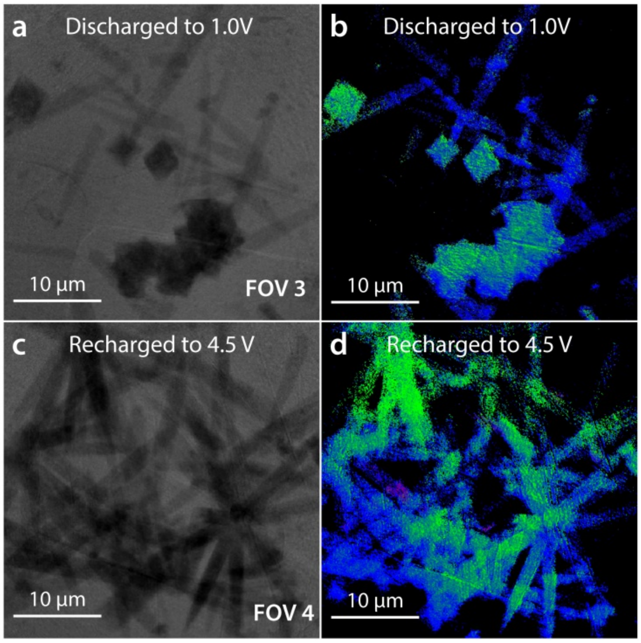

Supplementary Figure 7. Chemical phase maps generated from other areas of the same *operando* FeF_3 electrode (made of a mixture of MWs and polyhedral) at the end of discharge and charge. (**a**) and (**b**) are absorption-contrast image and the resulting chemical phase map, respectively, taken at the end of discharge. It is clear that the MWs reacted more completely than the polyhedra. (**c**) and (**d**) are absorption-contrast image and the resulting chemical phase map, respectively, taken at the end of charge. The recharge reaction was not complete. Metallic Fe and Fe^{2+} were found to co-exist at the end of recharge, indicating the reconversion must go through Fe^{2+}-containing intermediate(s) during recharge.

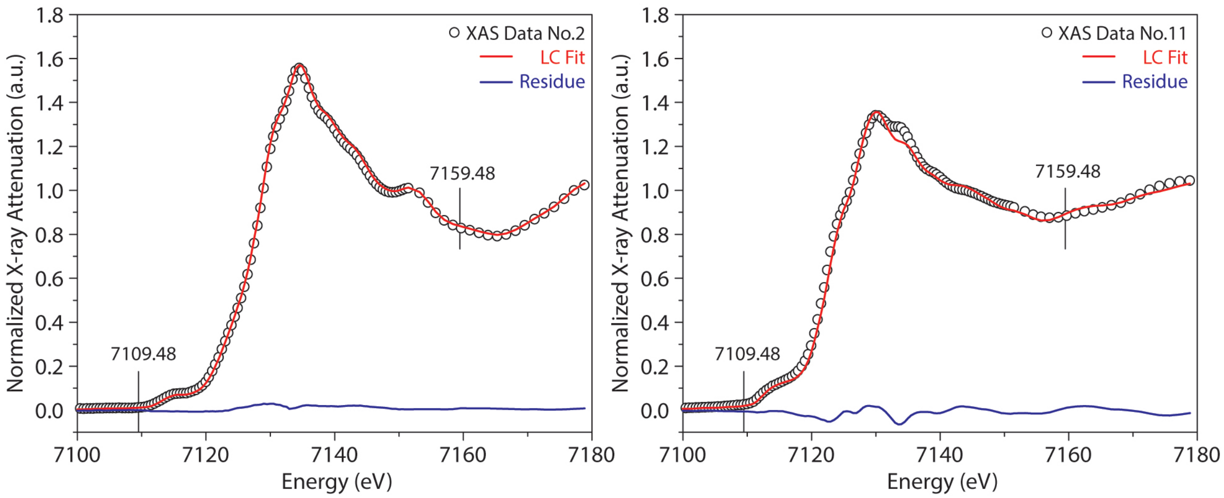

Supplementary Figure 8 | Two representative best fits of XAS spectra. The linear combinational fitting was performed in the energy range from 7109.48 to 7159.48 eV. The X-ray absorption data were processed and analyzed using IFEFFIT-ATHENA.

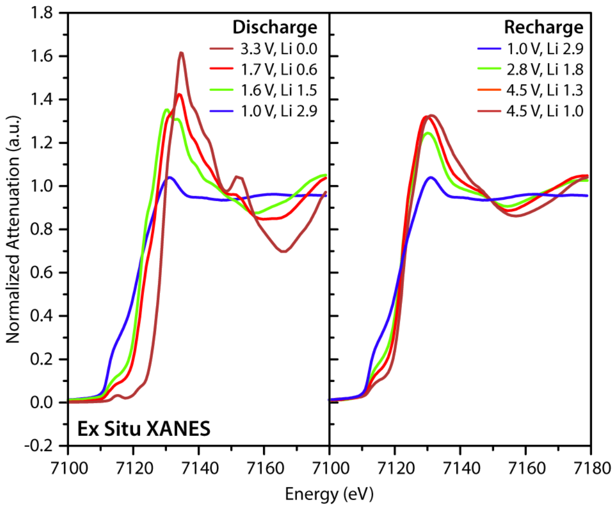

Supplementary Figure 9 | *Ex Situ* XAS on electrodes cycled to different states of discharge and charge. The electrodes were recovered from coin cells dissembled inside a glovebox and sealed between Kapton tapes.

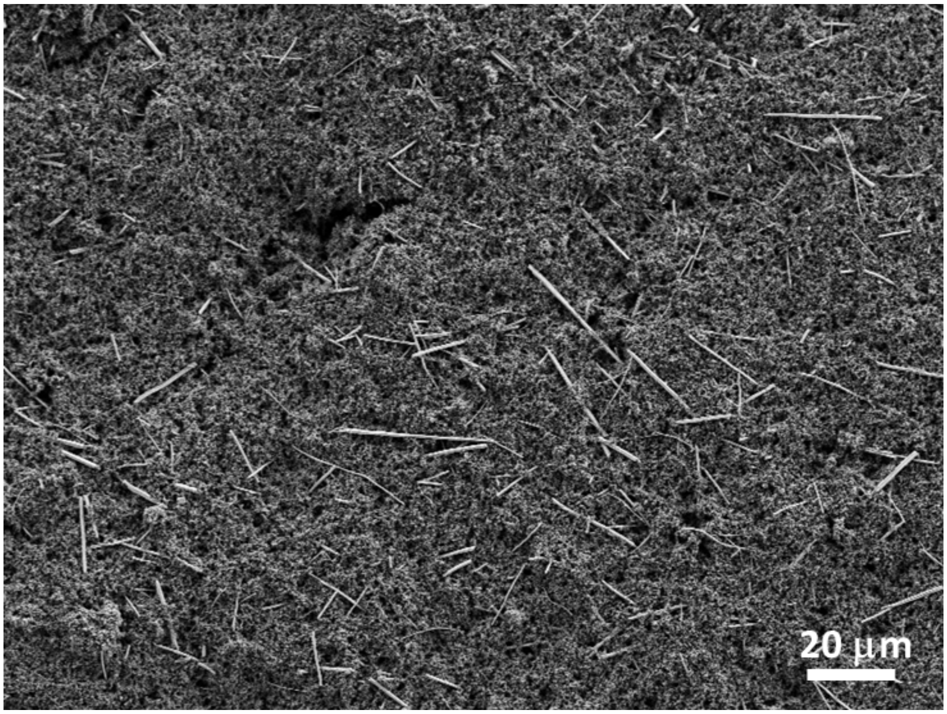

Supplementary Figure 10 | Representative SEM image of FeF_3 microwire electrode with binders and carbon black.

Supplementary Table

Supplementary Table 1 Details for the linear combinational fitting results in Figure 4d

XAS Data No.	R-factor	χ^2	$Fe^{3+}F_3$		$Fe^{2+}F_2$		Fe	
			Weight	Error	Weight	Error	Weight	Error
1	Did not fit. Pristine FeF_3 electrde							
2	0.0008	0.0184	0.8939	*0.0068*	0.1061	*0.0038*	NA, 2-standard fit	
3	0.0001	0.0013	0.7688	*0.0023*	0.1779	*0.0018*	0.0533	*0.0029*
4	0.0001	0.0024	0.6722	*0.0032*	0.2464	*0.0024*	0.0814	*0.0040*
5	0.0005	0.0065	0.6277	*0.0052*	0.2964	*0.0040*	0.0759	*0.0065*
6	0.0003	0.0058	0.4634	*0.0049*	0.3820	*0.0038*	0.1546	*0.0062*
7	0.0004	0.0086	0.4010	*0.0060*	0.4235	*0.0046*	0.1755	*0.0075*
8	0.0008	0.0140	0.3766	*0.0076*	0.4691	*0.0058*	0.1543	*0.0096*
9	0.0013	0.0295	0.2772	*0.0111*	0.5236	*0.0085*	0.1991	*0.0139*
10	0.0026	0.0614	0.2111	*0.0160*	0.6020	*0.0122*	0.1870	*0.0201*
11	0.0006	0.0127	0.2175	*0.0073*	0.5056	*0.0056*	0.2769	*0.0091*
12	0.0006	0.0081	0.2007	*0.0058*	0.4835	*0.0044*	0.3158	*0.0073*
13	0.0005	0.0108	0.1601	*0.0067*	0.5086	*0.0051*	0.3314	*0.0084*
14	0.0009	0.0186	0.1090	*0.0088*	0.5284	*0.0067*	0.3626	*0.0110*
15	0.0008	0.0164	0.0731	*0.0082*	0.5137	*0.0063*	0.4132	*0.0104*
16	0.0008	0.0156	0.0166	*0.0080*	0.4873	*0.0062*	0.4961	*0.0101*
17	0.0030	0.0396	0.0000	*0.0128*	0.4035	*0.0098*	0.5965	*0.0161*
18	0.0030	0.0330	0.0000	*0.0117*	0.2668	*0.0089*	0.7332	*0.0147*
19	0.0029	0.0276	0.0000	*0.0107*	0.1719	*0.0082*	0.8281	*0.0135*
20	0.0044	0.0387	0.0000	*0.0127*	0.1260	*0.0097*	0.8740	*0.0160*
21	0.0042	0.0361	0.0000	*0.0122*	0.1137	*0.0094*	0.8863	*0.0154*
22	0.0012	0.0113	0.0000	*0.0068*	0.1467	*0.0052*	0.8533	*0.0086*
23	0.0017	0.0168	0.0000	*0.0084*	0.1636	*0.0064*	0.8364	*0.0105*
24	0.0013	0.0134	0.0059	*0.0074*	0.1778	*0.0057*	0.8163	*0.0094*
25	0.0015	0.0201	0.0000	*0.0091*	0.2829	*0.0070*	0.7171	*0.0115*
26	0.0011	0.0181	0.0011	*0.0086*	0.3581	*0.0066*	0.6408	*0.0109*
27	0.0018	0.0333	0.0000	*0.0117*	0.4650	*0.0090*	0.5350	*0.0148*
28	0.0016	0.0316	0.0000	*0.0115*	0.5052	*0.0088*	0.4948	*0.0144*
29	0.0014	0.0312	0.0000	*0.0114*	0.5431	*0.0087*	0.4569	*0.0143*
30	0.0013	0.0275	0.0292	*0.0107*	0.5578	*0.0082*	0.4130	*0.0135*
31	0.0013	0.0291	0.0959	*0.0110*	0.6083	*0.0084*	0.2958	*0.0138*
32	0.0013	0.0312	0.1370	*0.0114*	0.6297	*0.0087*	0.2333	*0.0143*
33	0.0014	0.0253	0.1976	*0.0102*	0.5778	*0.0078*	0.2246	*0.0129*
34	0.0013	0.0249	0.2280	*0.0102*	0.5706	*0.0078*	0.2014	*0.0128*
35	0.0013	0.0234	0.2515	*0.0098*	0.5508	*0.0075*	0.1977	*0.0124*
36	0.0013	0.0219	0.2890	*0.0095*	0.5249	*0.0073*	0.1861	*0.0120*
37	0.0012	0.0227	0.3034	*0.0097*	0.5358	*0.0074*	0.1608	*0.0122*

- The linear combinational fitting was performed in the energy range from 7109.48 to 7159.48 eV.
- The rutile FeF_2 phase was used to represent all the possible rutile related Fe^{2+}-containing phases.

Supplementary Methods

Materials:

All chemicals were used as received without further purification. Hydrofluoric acid (aqueous HF solution, 48 wt%, CAS # 7664-39-3. *Warning: HF solution is highly corrosive and must be handled with care and proper protection. Please see the MSDS document in Sigma-Aldrich website*), iron (III) nitrate nonahydrate [$Fe(NO_3)_3{\cdot}9H_2O$, ≥98.0%, CAS # 7782-61-8], commercial iron trifluoride powder (FeF_3, ≥98.0%, CAS # 7783-50-8), iron difluoride powder (FeF_2, ≥98.0%, CAS # 7789-28-8), metallic iron particles (Fe, >99.0%, CAS # 7439-89-6), lithium ribbon (0.75 mm in thickness, >99.9%, CAS #7439-93-2) were purchased from Sigma Aldrich. Ethanol (200 proof, CAS #64-17-5) was purchased from Decon Laboratories Inc. N-methyl-2-pyrrolidone (NMP, ≥99.5%, CAS # 872-50-4), polyethylene separator membranes, polyvinylidene difluoride (PVDF, ≥99.5%, CAS # 24937-79-9) binder, lithium foils (15.6 mm in diameter × 0.45 mm in thickness, >99%, CAS #7439-93-2) and 2032-type coin cell cases were purchased from MTI Corporation. 1 M lithium hexafluorophosphate ($LiPF_6$) dissolved in 1:1 (v:v) ethyl carbonate (EC)/dimethyl carbonate (DMC) electrolyte was purchased from BASF. Carbon black [Super P® conductive, ≥99% (metal basis), CAS # 1333-86-4] and aluminum foils [0.025 mm in thickness, ≥99.45% (metal basis), CAS # 7429-90-5] were purchased from Alfa Aesar. Carbon Papers (Toray Paper 030 TGPH030-1005, 110 μm in thickness) were purchased from Fuel Cell Earth LLC.

Synthesis of FeF_3 Microwires and Polyhedra:

We developed the syntheses of two new FeF_3 (rhombohedral structure, $R\bar{3}c$) samples with different morphologies and microstructures by rationally controlling the supersaturation of growth solution[1–3]. In both syntheses, ethanol was used as the solvent, $Fe(NO_3)_3{\cdot}9H_2O$ as the Fe^{3+} source, and HF as the F^- source. Both F^- and H_2O act as ligands in complex with Fe^{3+} to control the supersaturation.

FeF_3 microwires (MWs) were prepared by thermal dehydration of α-$FeF_3 \cdot 3H_2O$ MWs in argon atmosphere. The precursor α-$FeF_3 \cdot 3H_2O$ MWs were synthesized *via* a new synthesis developed based on our previous work[1]. A diluted HF/ethanol solution (~5 M HF) was first prepared by mixing 1.812 mL HF aqueous solution (48 wt%) with 8.3 mL ethanol in a plastic centrifuge tube. Then, 7.0 mL ethanol, 1.0 mL diluted HF (~5 M), 1.0 mL deionized water were sequentially added into a 15 mL plastic centrifuge tube with caution. The centrifuge tube was sealed and gently shaken by hand with great caution to allow the liquids to mix. An Fe^{3+} solution in ethanol with a concentration of 532 mM was made by dissolving 1.616 g $Fe(NO_3)_3 \cdot 9H_2O$ in 7.5 mL ethanol. 1.000 mL of this Fe^{3+}/ethanol solution was then quickly injected into the HF/ethanol mixture using an Eppendorf pipet. The resulting colorless solution with a concentration ratio of $c(Fe^{3+}) : c(HF) : c(H_2O) \approx$ 53.2 mM: 500 mM: 11575 mM was gently mixed by shaking and kept in an oven at 60 °C for 18 h. No substrate was used during the reaction and aggressive agitation was avoided to suppress the formation of large β-$FeF_3 \cdot 3H_2O$ crystals. The white cloudy precipitate that appeared over time was collected by centrifugation at 5000 rpm for 5 min, washed with dry ethanol twice and vacuum-dried at room temperature. Typically, ~15 mg product of α-$FeF_3 \cdot 3H_2O$ MWs could be obtained from a 10 mL scale synthesis, corresponding to a reaction yield of ~17 % based on the limiting reagent of Fe. In order to make the anhydrous FeF_3 MWs, the as-synthesized white precipitate of α-$FeF_3 \cdot 3H_2O$ MWs was dehydrated in an inert atmosphere using a quartz tube reactor equipped with pressure and gas flow controls and placed a tube furnace (Lindberg/Blue M). Briefly, a large alumina boat containing ~150 mg α-$FeF_3 \cdot 3H_2O$ MWs were placed at the center of the tube furnace. The tube reactor was first slowly evacuated and flushed three times with argon gas and then the MWs were kept under vacuum (~40 mTorr) at 50 °C to for 20 min to dry completely. Then under a flowing argon gas at a flow rate of 100 sccm and a pressure of 770 Torr, the furnace temperature was slowly ramped from 50 °C to 350 °C in 150 min (~2 °C min^{-1}) and held at 350 °C for 30 min before naturally cooling down to produce a pale greenish/brownish product (~100 % reaction yield).

Rhombohedral phase FeF_3 polyhedra were prepared by thermal conversion of metastable cubic phase FeF_3 polyhedra, which were synthesized in ethanol solution. Briefly, a diluted HF/ethanol solution (~1 M HF) was first prepared by mixing 0.719 mL HF aqueous solution (48 wt%) with 19.3 mL ethanol in a plastic centrifuge tube.

Then, 7.5 mL ethanol, 1.0 mL diluted HF (~1 M), 0.5 mL deionized water were sequentially added into a 15 mL plastic centrifuge tube with caution. The centrifuge tube was sealed and gently shaken by hand with great caution to allow the liquids to mix. An Fe^{3+} solution in ethanol with a concentration of 200 mM was made by dissolving 0.808 g $Fe(NO_3)_3{\cdot}9H_2O$ in 10.0 mL ethanol. 1.000 mL of this Fe^{3+}/ethanol solution was then quickly injected into the HF/ethanol mixture using an Eppendorf pipet. The resulting colorless solution with a concentration ratio of $c(Fe^{3+}) : c(HF) : c(H_2O) \approx$ 20 mM: 100 mM: 4670 mM was gently mixed by shaking and kept in an oven at 60 °C for 24 h. No substrate was used during the reaction and aggressive agitation was avoided to suppress the formation of large β-$FeF_3{\cdot}3H_2O$ crystals. The light yellow precipitate that appeared over time was collected by centrifugation at 5000 rpm for 5 min, washed with dry ethanol twice and vacuum-dried at room temperature. Typically, 5–6 mg product of the cubic phase FeF_3 polyhedra could be obtained from a 10 mL scale synthesis, corresponding to a reaction yield of 22–27 % based on the limiting reagent of Fe. The as-synthesized cubic phase FeF_3 polyhedra were thermally annealed in an argon atmosphere at 350 °C for 30 min in the same quartz tube reactor described above to produce the rhombohedral phase FeF_3 polyhedra (~100 % reaction yield).

Material Characterization:

Scanning electron microscopy (SEM) images were acquired using a LEO 55 VP field emission scanning electron microscope at a working voltage of 5 kV. Transmission electron microscopy (TEM) images and electron diffraction (ED) patterns were acquired using either a Tecnai T-12 transmission electron microscope (120 kV) or a FEI Titan transmission electron microscope (200 kV). The TEM samples were prepared by dropcasting FeF_3 samples in ethanol onto lacey-carbon grids (Ted Pella Inc. lacey carbon type-A, 300 Mesh). Air/moisture-exposure was minimized as much as possible. Powder X-ray diffraction (PXRD) data were collected on a Bruker D8 diffractometer using Cu Kα radiation and corrected for background. The PXRD samples were prepared by drop-casting the ethanol suspensions of the samples onto glass substrates and dried. The Brunauer–Emmet–Teller

(BET) specific surface area and pore size distribution of the FeF_3 MWs were obtained from nitrogen adsorption–desorption isotherms measured by a Quantachrome Autosorb-1 gas sorption analyzer.

Processing of FeF_3 Cathode and Electrochemical Measurements:

Electrochemical measurements were performed on electrodes pasted on aluminum foils (~25 μm thickness), which were prepared from slurries containing 70 wt% active material, 20 wt% conductive carbon black and 10 wt% PVDF binder using NMP as the solvent. The mass loading of the active material (FeF_3) is around 2 mg cm^{-2}. CR2032-type coin cells were assembled in an argon-filled glovebox, using Li metal as the counter/quasi-reference electrode, 1 M $LiPF_6$ in EC/DMC (1/1 by volume) as the electrolyte, and electrolyte-soaked polyethylene films as the separator. All cells were aged a few hours before any tests. Electrochemical impedance spectroscopy (EIS) was performed using a Biologic SP-200 Potentiostat operated using EC-Lab software. EIS measurements were carried out over a frequency range from 200 kHz to 50 mHz with a perturbation voltage of 10 mV. Constant current discharge/charge tests were carried out using a Biologic VMP-3 potentiostat/galvanostat in a voltage window of 4.5–1.0 V at different current rates. All reported performance was calculated based on the mass of the active material.

Fabrication of Batteries for *Operando* Studies:

The *operando* experiments were performed using perforated 2032-type coin cells with ~4 mm holes on both sides of the cell cases. The holes were sealed using Kapton tapes. The holes need to be small to ensure a small cell impedance. The FeF_3 samples (polyhedra and MWs 1:1 by weight) were mixed with carbon black and PVDF binder in a weight ratio of 3:5:2 in NMP. The resulting slurry was pasted onto thin aluminum foils (~8 μm thickness) or carbon papers (~110 μm thickness) and then dried in vacuum to make electrodes. The thin aluminum foils and thin carbon papers are quite transparent to hard X-rays but still robust enough for handling, which is critical to the *operando* experiment. The density of samples on the electrodes was checked using an

optical microscope or a scanning electron microscope before packing into coin cells. The *operando* cells were assembled in an argon-filled glovebox using Li metal as the counter/quasi-reference electrode and polyethylene films soaked with 1 M $LiPF_6$ EC/DMC (1/1 by volume) electrolyte as the separator. No additional electrolyte was added during the cell packing in order to minimize the X-ray attenuation caused by the electrolyte liquid. The as-made cells were aged for a few hours and checked using electrochemical impedance spectroscopy before being used in the *operando* experiments, during which the cell was held by a custom-modified coin cell holder and discharged and charged at rate of ~1/15 C (1 C = 712 mA/g). The coin cell holder was purchased from MTI Corporation and attached to a stainless steel rod standing on the motorized stage. The positive lead of the cell holder was modified to make contact with the coin cell from the side so that the X-ray was not blocked. We also put a piece of perforated stainless steel foil between the positive lead and the coin cell to apply a gentle pressure to make better electrical contact.

*Operand*o Hard X-ray Spectro-Imaging:

The *operando* hard X-ray spectro-imaging experiments were performed using the full-field transmission X-ray microscope (FFTXM) at beamline X8c, National Synchrotron Light Source (NSLS), Brookhaven National Laboratory (BNL). The TXM operates using a bending magnetic source and a Si (111) double-crystal monochromator. It routinely operates in absorption contrast mode over a wide energy range from 5 to 11 keV with spatial and energy resolutions of ~25 nm and $\Delta E/E = 10^{-4}$, respectively.

In the *operando* experiment, the *operando* coin cell was mounted on an motorized X, Y, Z, θ stage and aligned so that the X-ray beam could transmit through the two holes in the cell. A field of view of $40 \times 40\ \mu m^2$ with a 2048 × 2048 CCD camera was used. The cell was continuously cycled in galvanostatic or potentiostatic mode and the X-ray absorption-contrast images (X-ray transmitted through the sample) and reference background images (X-ray passing through air) were collected in sequence under dynamic conditions. To track the phase transformations in the electrode, a full series of TXM images were collected at each states of discharge and charge. Each TXM image series was collected by scanning across the Fe *K*-edge (7112 eV) from 7091 to 7285 eV, with a

step size of 2 eV, and taking one TXM image at each energy step, which generated 1024 × 1024 XANES spectra with 2 × 2 binned pixels or 512 × 512 XANES spectra with 4 × 4 binned pixels depending on the choice of camera binning. The exposure time for each image was chosen depending on the intensity of the beam and was typically 8–10 seconds for 2 × 2 camera binning and 4–5 seconds for 4 × 4 camera binning during our experiments. Each chemical phase map took ~8 minutes to finish when using 4 × 4 camera binning and the output pixel size is ~80 nm.

Data Processing and Chemical Map Construction:

Each set of TXM images was first aligned using the Xradia Controller Software to correct the positioning errors of the motor stage, X-ray optics, or sample. Then all data were analyzed using a customized program developed in house using Matlab R2011b.

We first carried out background normalization for all TXM images using the unique reference background images (X-ray passing through air) collected at each energy to get the absorption-contrast images. One example is shown in Supplementary Figure1a, which is a field-of-view taken from an *operando* cell discharged to ~2.0 V. Then we could extract the XANES spectrum (X-ray attenuation versus energy raw data) at each pixel (1024 × 1024 pixels for binning 2, 512 × 512 pixels for binning 4). Here we used the single pixel spectrum (red open circles in Supplementary Figure1b) extracted from point S1 in Supplementary Figure1a to explain the data normalization procedures and compare the effectiveness of our methods with previously reported methods[4–7].

XANES spectra need to be normalized and scaled in order to be directly compared with each other and correctly fitted using standard reference spectra. The normalization is done using the data points in the pre-edge and post-edge regions[4–7], which involves five major steps: 1) Fit the pre-edge spectrum to a linear function and subtract it from the spectrum over the entire range of energy; 2) Identify the threshold energy E_0, which is the maximum of the 1st derivative of the spectrum; 3) Fit the post-edge spectrum to a linear function; 4) Determine the edge-jump value, which is the difference between the pre-edge function and post-edge function at the

threshold energy E_0; 5) Normalize the spectrum using the edge-jump value to make the pre-edge become ~0 and the post-edge become ~1.

The normalized spectrum can then be fitted to standard reference spectra to determine the ratio between different phases. This is because that based on Beer's Law, the total X-ray attenuation at each pixel can be considered as the sum of X-ray attenuation from each constituent phase with attenuation coefficient $\boldsymbol{\mu}$ and thickness $\boldsymbol{t}$, which can be written as:

$$-\ln(\boldsymbol{I_t}/\boldsymbol{I_0}) = \boldsymbol{\mu}(FeF_3) \cdot \boldsymbol{t}(FeF_3) + \boldsymbol{\mu}(FeF_2) \cdot \boldsymbol{t}(FeF_2) + \boldsymbol{\mu}(Fe) \cdot \boldsymbol{t}(Fe) \quad (1)$$

where $\boldsymbol{I_0}$ is the incident X-ray intensity and $\boldsymbol{I_t}$ is the X-ray intensity after it passes through the sample. Note that attenuation coefficient $\boldsymbol{\mu}$ and $-\ln(\boldsymbol{I_t}/\boldsymbol{I_0})$ are energy dependent [as the $-\ln(\boldsymbol{I_t}/\boldsymbol{I_0})$ versus *energy* plot is the XANES spectrum] and the rutile FeF_2 phase was used to represent all the possible rutile related Fe^{2+}-containing phases. This is a reasonable approximation because it was reported that the Li_xFeF_3 (when $x \approx 1.0$) phase contains structural features that are found in the rutile FeF_2 structure (*23*). As other battery components in the pathway of the beam (such as electrolyte, carbon, PVDF binder, and separator) also attenuate the X-ray during the *operando* experiment, their contribution (denoted by $\boldsymbol{A_{bkg}}$) is non-trivial and should also be taken into consideration. The modified equation can be written as:

$$-\ln(\boldsymbol{I_t}/\boldsymbol{I_0}) = \boldsymbol{\mu}(FeF_3) \cdot \boldsymbol{t}(FeF_3) + \boldsymbol{\mu}(FeF_2) \cdot \boldsymbol{t}(FeF_2) + \boldsymbol{\mu}(Fe) \cdot \boldsymbol{t}(Fe) + \boldsymbol{A_{bkg}} \quad (2)$$

Previous methods depend heavily upon the strong X-ray absorption of large-sized samples (10–20 μm) and considered the X-ray absorption of the materials under study approximately equal to the total X-ray absorption[4–7]. This approximation is no longer valid for the smaller and weakly absorbing samples investigated herein. As a result, those methods failed to correctly normalize the XANES spectra for smaller and less X-ray absorbing samples, such as the porous FeF_3 MWs examined herein (Supplementary Figure1a). An example of such improperly normalized spectra is shown in Supplementary Figure1e, which is clearly off the scale compared with the standard reference spectra (Supplementary Figure2). When the fitting was carried out using these improperly normalized spectra at all the pixels, the quality of the resulting chemical phase map is unsatisfactory (see

Supplementary Figure1b), because very few pixels could be fitted correctly to pass the *R-value* filter (misfit filter).

We solved this normalization problem by approximating the internal background X-ray absorption ($\boldsymbol{A_{bkg}}$, black circles in Supplementary Figure1d) using the X-ray attenuating information readily available from the area that does not contain the FeF_3 sample but all the other components in the *operando* cell, such as electrolyte, polymeric binder, carbon black, current collector, and separator (the black box in Supplementary Figure1a). We first subtracted the internal background spectrum (black circles in Supplementary Figure1d) from the total X-ray attenuation (red circles in Supplementary Figure1d) and then carried out the data normalization following the aforementioned five-step procedure, which yielded correctly normalized spectrum as shown in Supplementary Figure1f. Using our custom-developed program, this normalization procedure could be conveniently applied to the spectra at all pixels.

The correctly normalized spectrum at each pixel was then fitted with the linear combination of three $\boldsymbol{\mu t}$ values. The ratio of the weighing factor is an analogue of the thickness fraction and therefore represents the volume fractions of solid state phases containing different Fe oxidation states. The fitting was carried out by minimizing the $\boldsymbol{R}$ value (a measure of misfit) for each spectrum at each pixel, which is defined as:

$$\boldsymbol{R} = \sum_{\boldsymbol{Ei}}^{\boldsymbol{Ef}} (\boldsymbol{dataE} - \boldsymbol{refE})^2 / \sum_{\boldsymbol{Ei}}^{\boldsymbol{Ef}} \boldsymbol{dataE}^2 \qquad (3)$$

where $\boldsymbol{Ei}$ is 7091 eV, $\boldsymbol{Ef}$ is 7285 eV, $\boldsymbol{dataE}$ is the normalized spectrum at each pixel for the given energy $\boldsymbol{E}$, and $\boldsymbol{refE}$ is the possible fitting reference value that is a linear combination of X-ray attenuation of FeF_3, FeF_2, and Fe. The standard reference XANES spectra were collected from commercial FeF_3, FeF_2, and Fe powders under the same conditions using TXM and verified using a standard spectroscopy beamline X18A at NSLS, BNL. During the fitting, we considered all possible combinations with FeF_3, FeF_2, and Fe with 2 vol% resolution, which was a total of 1326 possible combinations. *R values* were minimized at each pixel to find the best-matched phase combination of different Fe oxidation states so that Red-Green-Blue (Red: Fe^{3+}, Green: Fe^{2+}, Blue: Fe) colors can be assigned accordingly to generate the chemical phase map (Supplementary Figure1c). We applied an *R-value*

filter (misfit filter) to the resulting phase map and only pixels with $R < 0.08$ were displayed in order to give the most accurate chemical phase information. The comparison between Supplementary Figure1c and S1b clearly show that our new data processing procedures could generate a higher quality chemical phase map compared with the previously reported methods. These new data processing procedures were consistently employed to yield the chemical phase maps shown in Figure 2 and 3 in the manuscript text and Supplementary Figure 6 and 7 in the Supplementary Information.

Operando X-ray Absorption Spectroscopy Experiment on FeF_3 Microwires*:* The *operando* X-ray absorption experiments were performed at beamline X18A, National Synchrotron Light Source, Brookhaven National Laboratory. The measurements were performed in transmission mode using a Si (111) double-crystal monochromator, which was detuned to ~35% of its original maximum intensity to eliminate the high order harmonics in the beam. A reference X-ray absorption spectrum of Fe (*K*-edge 7112 eV) was simultaneously collected using a standard Fe foil. Energy calibration was done using the first inflection point of the Fe *K*-edge spectrum as the reference point. The X-ray absorption data were processed and analyzed using IFEFFIT-ATHENA. Standard reference spectra from commercial FeF_3, FeF_2, and Fe powders were also collected in order to carry out linear combination analysis to determine the ratio between different Fe oxidation states. The rutile FeF_2 phase was used to represent all the possible rutile related Fe^{2+}-containing phases. This is a reasonable approximation because it was reported that the Li_xFeF_3 (when $x \approx 1.0$) phase contains structural features that are found in the rutile FeF_2 structure[S8].

The *operando* experiments were performed using perforated 2032-type coin cells with holes on both sides of the cell cases. The holes were sealed using Kapton tapes. The FeF_3 MWs were mixed with carbon black and PVDF binder in a weight ratio of 7:2:1 in NMP. The resulting slurry was pasted onto thin aluminum foils (~25 μm thickness) to make electrodes. The *operando* cells were assembled in an argon-filled glovebox using Li metal as the counter/quasi-reference electrode and polyethylene films soaked with 1 M $LiPF_6$ EC/DMC (1/1 by volume) electrolyte as the separator. The as-made cells were aged for a few hours and checked using electrochemical

impedance spectroscopy before being used in the *operando* experiments, during which the cell was discharged and charged at rate of ~1/12 C (1 C = 712 mA/g).

Supplementary References

1. Li, L., Meng, F., Jin, S., High-capacity lithium-ion battery conversion cathodes based on iron fluoride nanowires and insights into the conversion mechanism. *Nano Lett.* **12,** 6030-6037 (2012).

2. Morin, S. A., Bierman, M. J., Tong, J., Jin, S., Mechanism and kinetics of spontaneous nanotube growth driven by screw dislocations. *Science* **328,** 476-480 (2010).

3. Meng, F., Morin, S. A., Forticaux, A., Jin, S., Screw dislocation driven growth of nanomaterials. *Acc. Chem. Res.* **46,** 1616-1626 (2013).

4. Wang, J., Chen-Wiegart, Y.-c. K., Wang, J., In Situ chemical mapping of a lithium-ion battery using full-field hard X-ray spectroscopic imaging. *Chem. Commun.* **49,** 6480-6482 (2013).

5. Yang, F., et al., Nanoscale morphological and chemical changes of high voltage lithium–manganese rich NMC composite cathodes with cycling. *Nano Lett.* **14,** 4334-4341 (2014).

6. Wang, J., Chen-Wiegart, Y.-c. K., Wang, J., In operando tracking phase transformation evolution of lithium iron phosphate with hard X-ray microscopy. *Nature Commun.* **5,** 4570 (2014).

7. Yu, Y.-S., et al., Nonequilibrium pathways during electrochemical phase transformations in single crystals revealed by dynamic chemical imaging at nanoscale resolution. *Adv. Energy Mater.* Early View, DOI: 10.1002/aenm.201402040 (2014).

8. Yamakawa, N., Jiang, M., Key, B., Grey, C. P., Identifying the local structures formed during lithiation of the conversion material, iron fluoride, in a Li-ion battery: A solid-state NMR, X-ray diffraction, and pair distribution function analysis study. *J. Am. Chem. Soc.* **131,** 10525-10536 (2009).S
